# Suprachoroidal spheroidal mesenchymal stem cell implantation in retinitis pigmentosa: clinical results of 6 months follow-up

**DOI:** 10.1186/s13287-023-03489-z

**Published:** 2023-09-13

**Authors:** Berna Özkan, Büşra Yılmaz Tuğan, Cansu Hemşinlioğlu, Gözde Sır Karakuş, Özlem Şahin, Ercüment Ovalı

**Affiliations:** 1 Department of Ophthalmology, Acıbadem Mehmet Ali Aydınlar University, Istanbul, Turkey; 2 Department of Ophthalmology, Kocaeli University, Kocaeli, Turkey; 3 Acıbadem Labcell Cellular Therapy Center, Istanbul, Turkey; 4 Department of Ophthalmology, Marmara University, Istanbul, Turkey

**Keywords:** Retinitis pigmentosa, Mesenchymal stem cells, Spheroids, Suprachoroidal implantation

## Abstract

**Purpose:**

This prospective clinical case series aimed to evaluate the effect of suprachoroidal implantation of mesenchymal stem cells (MSCs) in the form of spheroids as a stem cell therapy for retinitis pigmentosa (RP) patients with relatively good visual acuity.

**Methods:**

Fifteen eyes of 15 patients with RP who received suprachoroidal implantation of MSCs in the form of spheroids were included. Best-corrected visual acuity (BCVA), 10–2 and 30–2 visual field examination and multifocal electroretinography (mfERG) recordings were recorded at baseline, postoperative 1st, 3rd and 6th months during follow-up.

**Results:**

Baseline median BCVA of RP patients was 1.30 (1.00–2.00) logMAR. BCVA has improved to 1.00 (0.50–1.30), 0.80 (0.40–1.30) and 0.80 (0.40–1.30) at the postoperative 1st, 3rd and 6th months, respectively. The improvements from baseline to the 3rd and 6th months were statistically significant (*p* = 0.03 and *p* < 0.001, respectively). In the 30–2 VF test, median MD was significantly improved at the 6th month compared to baseline (*p* = 0.030). In the 10–2 VF test, the median MD value was significantly different at the 6th month compared to the baseline (*p* = 0.043). The PSD value of the 10–2 VF test was significantly different at the 6th month compared to the 3rd month (*p* = 0.043). The amplitudes of P1 waves in < 2°, 5°–10° and 10°–15° rings improved significantly at the postoperative 6th month (*p* = 0.014, *p* = 0.018 and *p* = 0.017, respectively). There was also a statistically significant improvement in implicit times of P1 waves in 10°–15° ring at the postoperative 6th month (*p* = 0.004).

**Conclusion:**

Suprachoroidal implantation of MSCs in the form of spheroids as a stem cell therapy for RP patients with relatively good visual acuity has an improving effect on BCVA, VF and mfERG recordings during the 6-month follow-up period. Spheroidal MSCs with enhanced effects may be more successful in preventing apoptosis and improving retinal tissue healing in RP patients.

## Introduction

Retinal degenerative disorders are a group of diseases known for the progressive loss of retinal cells. Retinitis pigmentosa (RP) is the most common hereditary retinal disease that comes under the heading of retinal degenerative disorders. Cumulative damage of the retinal cells causes nyctalopia and peripheral visual field loss in RP. The progression of the disease damages the central photoreceptors in the late phase. The disease does not affect only photoreceptors. The damage in the photoreceptors affects the surrounding cell layers in the retina, retinal pigment epithelium (RPE) and choroid [1, 2].

At present, there is no treatment that cures RP. Gene therapy is the most promising treatment; however, hundreds of genes cause the disease. Mesenchymal stem cell (MSC) treatment has recently been studied as a viable alternative for different retinal diseases [3–9]. MSCs have been shown to have significant paracrine and immunomodulatory properties by secretion of trophic factors stimulating RPE or similar to those produced by RPE [10–19]. Animal studies demonstrated that MSCs are effective in suppressing chronic inflammation, preventing retinal degeneration, rescuing photoreceptors and preventing apoptosis in neurodegenerative and ischemic retinal disorders [20–26].

The current study aims to evaluate the effect of suprachoroidal implantation of MSCs in the form of spheroids as a stem cell therapy of RP on visual acuity, visual field analysis and electrophysiological testing to reveal potential functional and anatomical impacts of the therapy.

## Methods

A prospective, non-randomized, phase I/II clinical trial was conducted in patients with RP at the Department of Ophthalmology, Acibadem University, Medical School (ClinicalTrials.gov Identifier: NCT05712148). The study was approved by the ethics committee of Review Board of Cell, Organ and Tissue Transplantation Department of Turkish Ministry of Health (56,733,164/203 E.3858). The study was performed in accordance with the Declaration of Helsinki. Since the central ethics committee requires individual approval, individual consent was obtained.

### Patient evaluation

Fifteen patients with clinical and genetic diagnoses of RP were included in the study. Patients underwent ophthalmic examination including best-corrected visual acuity (BCVA), intraocular pressure, anterior segment examination and dilated fundus examination (with topical tropicamide 1% and phenylephrine 2.5%). Each eye underwent spectral-domain OCT scanning with Cirrus 5000 HD-OCT Angioplex (Carl Zeiss Meditec, Dublin, CA, USA), fundus autofluorescence and fundus fluorescein angiography (Heidelberg Engineering, Germany). MD (mean deviation) and PSD (pattern standard deviation) parameters of 10–2 and 30–2 visual field (VF) testing strategies with a Humphrey Field Analyzer model 750I (Carl Zeiss Meditec, Dublin, CA, USA) were obtained. The electrophysiological function was assessed with mfERG evaluation (MonPack 3, Metrovision, France) according to the International Society for Clinical Electrophysiology of Vision (ISCEV) guidelines [27]. BCVA was converted to the logarithm of the minimal angle of resolution (logMAR) equivalent.

The patients were excluded from the study, if they had (1) coexisting ocular pathology that may affect visual acuity, visual field and retinal morphology such as glaucoma, uveitis and previous vitreoretinal surgery, (2) coexisting cataract that may affect mfERG, visual field and/or ocular imaging, (3) refractive error that may affect measurements higher than + 6.00D and lower than − 6.00D, (4) coexisting systemic diseases that may affect visual function such as diabetes, vasculitis, rheumatological diseases and chronic immunosuppressive use, (5) periocular injection of platelet-rich plasma (PRP) and transcorneal electrical stimulation in the previous 6 months and (6) previous ocular surgery.

### Electrophysiologic testing

After 30 min of dark adaptation and pupil dilatation with the application of one drop of tropicamide 1% (Tropamid, Bilim ˙Ilaç, Turkey), phenylephrine 2.5% (Mydfrin, Alcon) and proparacaine hydrochloride 0.5% (Alcaine, Alcon), ERG jet electrodes were placed. Multifocal electroretinographies were recorded after pupil dilatation. The stimulated retinal area was subtended in an area of 60° × 55°; 61 hexagon stimulants were used with alternating black (5 cd/m^2^) and white (100 cd/m^2^) stimulants. The concentric rings were analyzed according to International Society for Clinical Electrophysiology of Vision standards [13]. The amplitude and latencies of P1, N1 and N2 components were recorded for every ring. The mean signal amplitudes (MSAs) of multifocal electroretinography (mfERG) in the macula (central 0°–2°) and the peripheral (2°–5°, 5°–10°, 10°–15° and > 15°) signal amplitude changes were evaluated separately.

### Spheroidal stem cell preparation

First passage umbilical cord-derived mesenchymal stem cell was obtained from Labcell Cellular Laboratory Center (Acibadem Labmed, Acibadem University, Istanbul) which provides GMP (good manufacturing practices) conditions.

### Spheroid production

The spheroid production continued in GMP condition. First passage umbilical cord-derived mesenchymal stem cells were used in the production of spheroids. A total of 100,000 mesenchymal stem cells were suspended with 100 μl. Serum-free medium (MSC NutriStem^®^ XF Medium, Sartorius) containing 1% ciprofloxacin (Polipharma). Each well of the low attachment 96-well plate was seeded with 100,000 cells in 100-μl medium. The cells were incubated at 37 °C for 48 h. At the end of 48 h, all spheroids were collected with a micropipette and transferred into Ringer's lactate solution (Osel/Biofleks) containing 1% HSA (CSL Behring). Spheroids were washed 3 times with Ringer’s lactate solution containing 1% HAS (CSL Behring). Fifty spheroids were produced for one patient (50 spheroids containing 5 × 10^6^ cells were produced). Five of 50 spheroids were reserved for quality control analysis. The remaining 45 spheroid membranes were embedded in the matrix.

### Matrix production and cell embedding culture

**Fig. 1:**
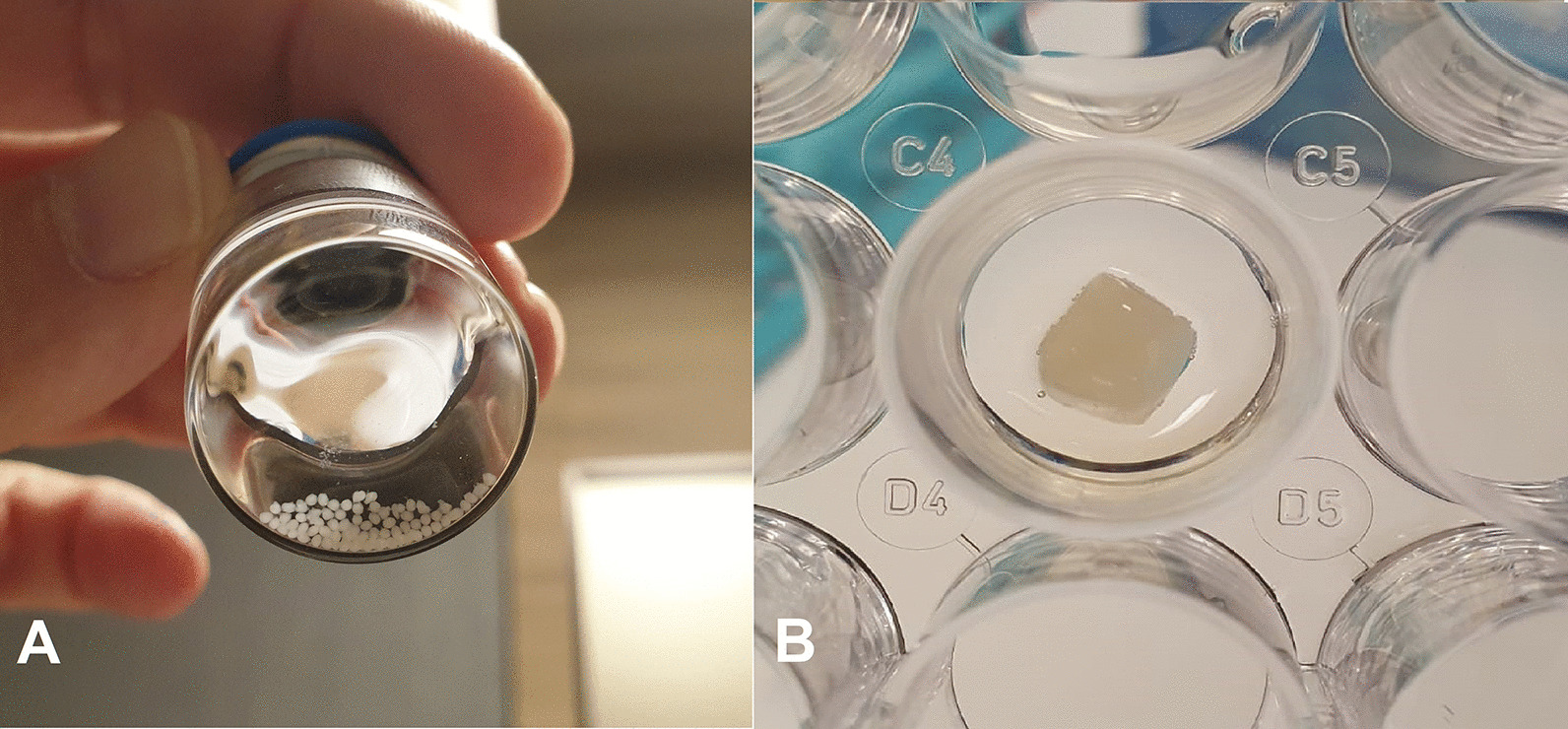
**A**. Representative image of spheroids. **B**. Representative image of spheroids embedded in the middle of the matrix transferred into Ringer's lactate solution (Osel/Biofleks) containing 1% HAS (CSL Behring) and transferred to the operating room in this solution

Matrix mixture containing 225-µl cryoprecipitate + 22.5-µl calcium (Adeka) + 2.5-µl transamine (Haver) was added to each well of the 96-well plates. When the matrix became semi-solid, 45 spheroids were embedded in the middle of the matrix and incubated at 37 °C for 45 min. The matrix, which became completely solid after 45 min, was removed with the help of a scalpel, transferred into Ringer's lactate solution (Osel/Biofleks) containing 1% HAS (CSL Behring) and transferred to the operating room in this solution (Fig. 1)*.*

### Quality control analysis

Microbiological blood culture, fungal, endotoxin analysis and purity (ciprofloxacin < 0.1 µg/ml), efficiency (adipocyte and cartilage differentiation analysis) cell count/viability and flow cytometric analysis (CD34 < %2, CD45 < %4, CD90 > %80, HLA-DR < %4, CD105 > %60 and CD73 > %70) were studied from the reserved spheroids for quality control analyses.

### Surgical technique

**Fig. 2:**
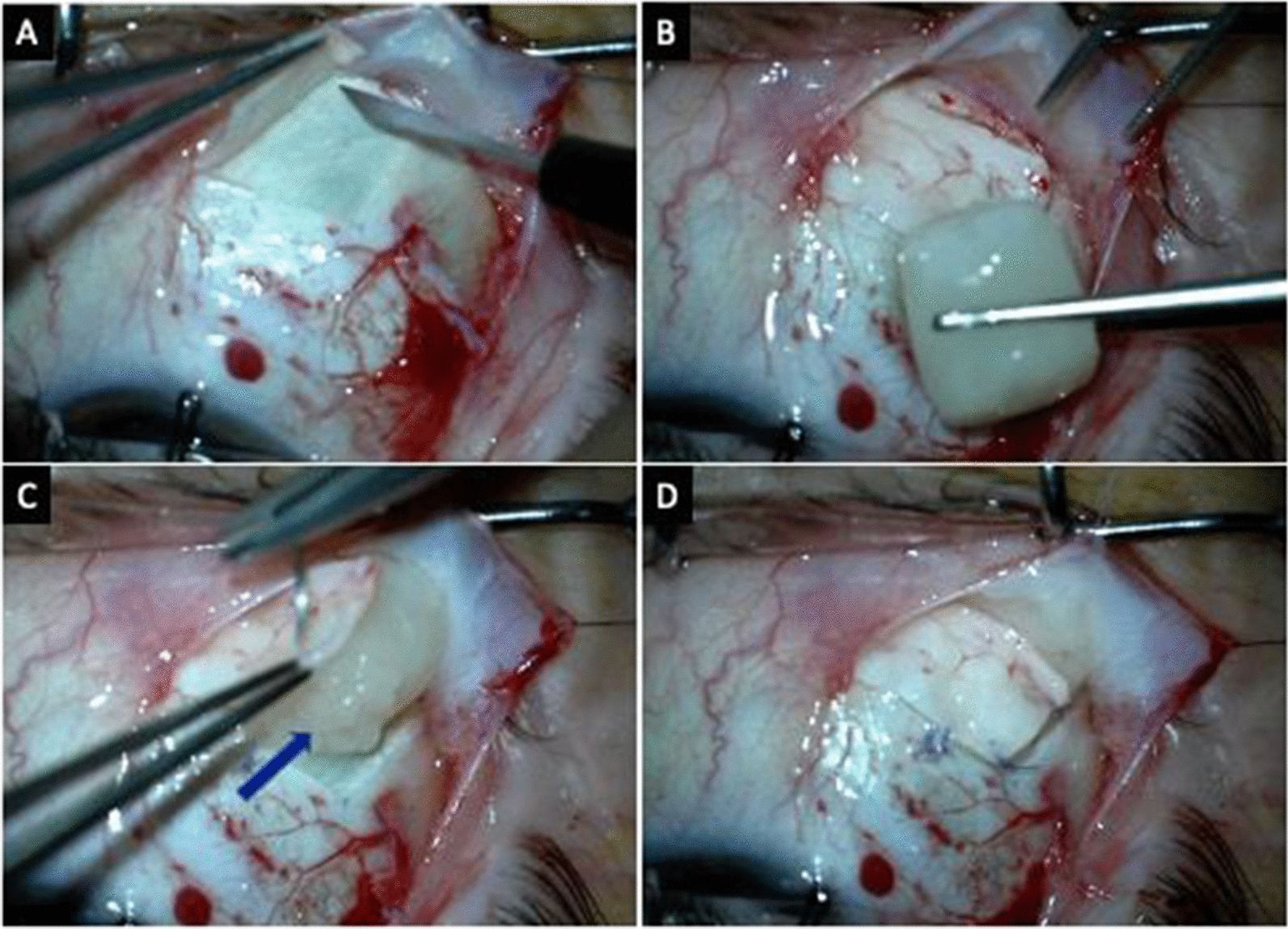
**a** Preparation of the scleral bed. **b** Matrix containing the spheroids. **c** Matrix placed in the scleral bed. **d** After suturing the flap over the matrix, the sutures are placed tightly in anterior edge of the flap and loosely in the free edge and posterior edge

Patients underwent surgery under general anesthesia. The inferotemporal quadrant was chosen as the surgical area, and a 6.0 Silk was used as the anchoring suture near the limbus conjunctiva, and tenon was opened as 6-mm long cut at 6 mm from limbus parallel to the limbus and the edges of the cut were advanced 3 mm posteriorly. Two 8.0 Vicryl sutures were used as traction sutures at the anterior corners of the conjunctiva. The tenon was dissected over the sclera posteriorly. Then, we performed a 7 × 7 scleral flap. Anterior margin of the scleral flap was created at 8 mm from the limbus, parallel to the limbus with 30 degrees ophthalmic knife. Two other half-thickness side cuts were made to create a U-shaped flap that has its base parallel to the lateral rectus muscle. Then starting from the inferoanterior edge, a deep scleral flap was dissected with a crescent blade. During the dissection, black choroidal reflex should be observed all around the surface of the flap bed (Fig. 2A). The fibrin plug carrying spheroidal stem cells was placed over the choroid that was seen under the thin sclera (Fig. 2B and C). It was covered by the scleral flap, and the flap was sutured to its original position from its edges with a 7.0 Vicryl suture (Fig. 2D). Tenon and conjunctiva were closed separately with an 8.0 Vicryl suture.

### Statistical analysis

IBM SPSS for Windows version 20.0 (SPSS, Chicago, IL, USA) was used to perform all statistical analyses. The assumption of normality was tested with Shapiro–Wilk’s test. Continuous variables with normal distribution were presented as mean ± standard deviation, and continuous variables with no normal distribution were presented as median (25–75th percentile). Counts (percentages) represented categorical variables. Pre- and post-injection comparisons were analyzed by paired samples t-test/Friedman’s two-way ANOVA test, whichever was appropriate. Dunn’s test was used for the pairwise multiple comparisons. All statistical analyses were performed with 5% significance, and a two-sided *p*-value < 0.05 was considered statistically significant.

## Results

Fifteen eyes of 15 patients were evaluated in the study. The mean age of the patients was 38.6 ± 12.2 years. Six patients were female, and nine patients were male.

**Fig. 3:**
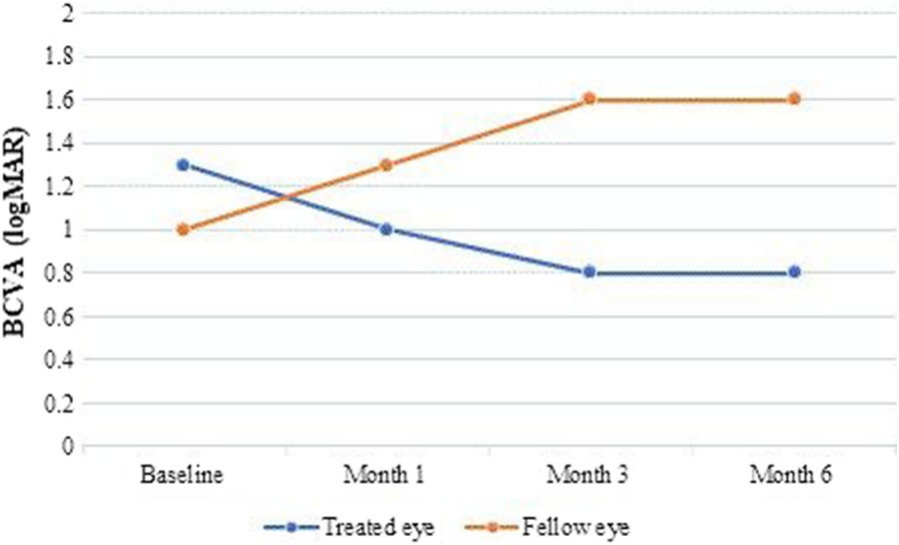
Trends of BCVA of treated and fellow eyes during the follow-up

The demographic and clinical features of the 15 study patients are shown in Table 1. The median BCVA (logMAR) of treated eyes was 1.30 (1.00–2.00) at baseline examination. During the follow-up, BCVA has improved to 1.00 (0.50–1.30), 0.80 (0.40–1.30) and 0.80 (0.40–1.30) at the postoperative 1st, 3rd and 6th months, respectively. The improvements from baseline to the 3rd and 6th months were statistically significant (*p* = 0.03 and *p* < 0.001, respectively). No significant change was observed in BCVA of the fellow eyes at the postoperative 1st, 3rd and 6th months compared to the baseline (*p* > 0.05, for all) (Table 2) (Fig. 3). While no significant difference was found between treated eyes and fellow eyes regarding baseline, month 1, month 3 BCVA values and month 6 BCVA was significantly improved in treated eyes compared to fellow eyes (*p* = 0.039).

**Table 1:** Demographic and visual acuity results according to ETDRS letters and logMAR equivalent of enrolled subjects

No./age (years)/sex/eye	Baseline BCVA (ETDRS letters/logMAR)	1-month BCVA (ETDRS letters/logMAR)	3-month BCVA (ETDRS letters/logMAR)	6-month BCVA (ETDRS letters/logMAR)
1/40/female/right	20/1.3	35/1.0	50/0.7	60/0.5
2/39/male/right	20/1.3	20/1.3	20/1.3	20/1.3
3/24/male/left	35/1.0	60/0.5	65/0.4	65/0.4
4/50/female/right	35/1.0	35/1.0	35/1.0	45/0.8
5/56/male/right	35/1.0	60/0.5	60/0.5	60/0.5
6/10/female/right	35/1.0	45/0.8	45/0.8	45/0.8
7/51/female/left	–/2.0	–/2.0	–/1.7	10/1.5
8/49/female/left	–/3.0	–/2.0	–/1.7	–/1.7
9/32/male/right	65/0.4	70/0.3	75/0.2	75/0.2
10/52/male/left	20/1.30	60/0.5	60/0.5	60/0.5
11/34/female/right	–/2.0	–/2.0	–/2.0	–/2.0
12/34/male/right	–/2.0	20/1.3	20/1.3	20/1.3
13/36/male/right	–/2.0	20/1.3	20/1.3	20/1.3
14/34/male/right	50/0.7	65/0.4	65/0.4	70/0.3
15/35/male/left	50/0.7	60/0.5	65/0.4	75/0.2

*BCVA* best-corrected visual acuity, *ETDRS* early treatment for diabetic retinopathy study and *logMAR* logarithm of minimum angle of resolution

**Table 2:** BCVA and visual field examination data of retinitis pigmentosa (RP) patients at baseline, 1 month, 3 months and 6 months

		Baseline	Month 1	Month 3	Month 6	p*
**BCVA logMAR**						
Treated eye	Median (IQR)	1.30 (1.00–2.00)^a^	1.00 (0.50–1.30)^ab^	0.80 (0.40–1.30)^b^	0.80 (0.40–1.30)^b^	** < 0.001**
	Mean ± SD	1.38 ± 0.68	1.02 ± 0.60	0.94 ± 0.56	0.89 ± 0.58	
Fellow eye	Median (IQR)	1.00 (0.70–2.00)	1.30 (0.50–2.00)	1.60 (0.60–2.50)	1.60 (0.70–2.50)	0.152
	Mean ± SD	1.42 ± 0.91	1.38 ± 0.96	1.56 ± 1.02	1.59 ± 1.00	
***p***		0.888**	0.413***	0.068**	0.039**	
**MD 30/2**						
Treated eye	Median (IQR)	32.2 (30.4–32.8)^a^	31.5 (29.1–32.6)^ab^	31.6 (19.9–32.4)^ab^	30.6 (27.1–32.5)^b^	**0.029**
	Mean ± SD	31.2 ± 3.3	30.8 ± 2.0	31.0 ± 1.9	28.2 ± 8.0	
Fellow eye	Median (IQR)	32.3 (30.3–33.8)	31.9 (30.3–33.6)	32.4 (30.4–33.8)	32.2 (31.3–34.7)	0.985
	Mean ± SD	32.0 ± 2.1	31.9 ± 2.3	32.3 ± 1.9	32.6 ± 1.8	
***p***		0.829***	0.215**	0.132**	0.051***	
**PSD 30/2**						
Treated eye	Median (IQR)	3.4 (1.6–5.6)	4.0 (2.1–6.0)	4.8 (2.7–5.6)	4.6 (2.7–5.6)	0.218
	Mean ± SD	3.7 ± 1.8	4.1 ± 1.9	4.1 ± 1.7	4.5 ± 1.9	
Fellow eye	Median (IQR)	3.7 (1.7–6.2)	3.8 (1.5–5.4)	3.5 (1.8–5.6)	3.3 (2.0–4.9)	0.682
	Mean ± SD	3.7 ± 2.2	3.5 ± 2.0	3.8 ± 2.4	3.6 ± 2.0	
***p***		0.997**	0.465**	0.753**	0.300**	
**MD 10/2**						
Treated eye	Median (IQR)	27.7 (25.2–34.3)^a^	26.3 (21.8–34.3)^ab^	26.1 (21.0–34.2)^ab^	28.7 (21.6–34.3)^b^	**0.027**
	Mean ± SD	28.7 ± 5.0	27.7 ± 5.7	27.5 ± 6.0	28.0 ± 6.6	
Fellow eye	Median (IQR)	30.8 (22.0–32.1)	30.9 (23.8–32.7)	31.6 (27.2–32.9)	30.0 (25.9–31.6)	0.177
	Mean ± SD	28.0 ± 5.0	28.6 ± 5.0	30.3 ± 3.8	29.1 ± 2.6	
***p***		0.882***	1.000***	0.417***	1.000***	
**PSD 10/2**						
Treated eye	Median (IQR)	5.5 (3.0–6.1)^ab^	4.7 (2.3–6.0)^ab^	4.8 (1.5–5.3)^a^	5.0 (1.7–6.3)^b^	**0.024**
	Mean ± SD	4.8 ± 2.0	4.2 ± 2.0	3.9 ± 2.3	4.3 ± 2.3	
Fellow eye	Median (IQR)	5.7 (4.2–6.1)	5.2 (4.1–6.3)	5.0 (3.5–6.0)	4.9 (3.5–6.7)	0.896
	Mean ± SD	5.1 ± 1.7	5.1 ± 1.7	4.5 ± 1.6	4.7 ± 2.0	
***P***		0.882***	0.331***	0.470***	0.867***	

*BCVA* best-corrected visual acuity, *MD* mean deviation (dB), *PSD* pattern standard deviation (dB), *SD* standard deviation and *IQR* interquartile range

*Friedman’s two-way ANOVA test

**Independent samples *t*-test

***Mann–Whitney *U*-test

Medians followed by similar lower case letters are not significantly different

Bold face values represent statistical significance

**Fig. 4:**
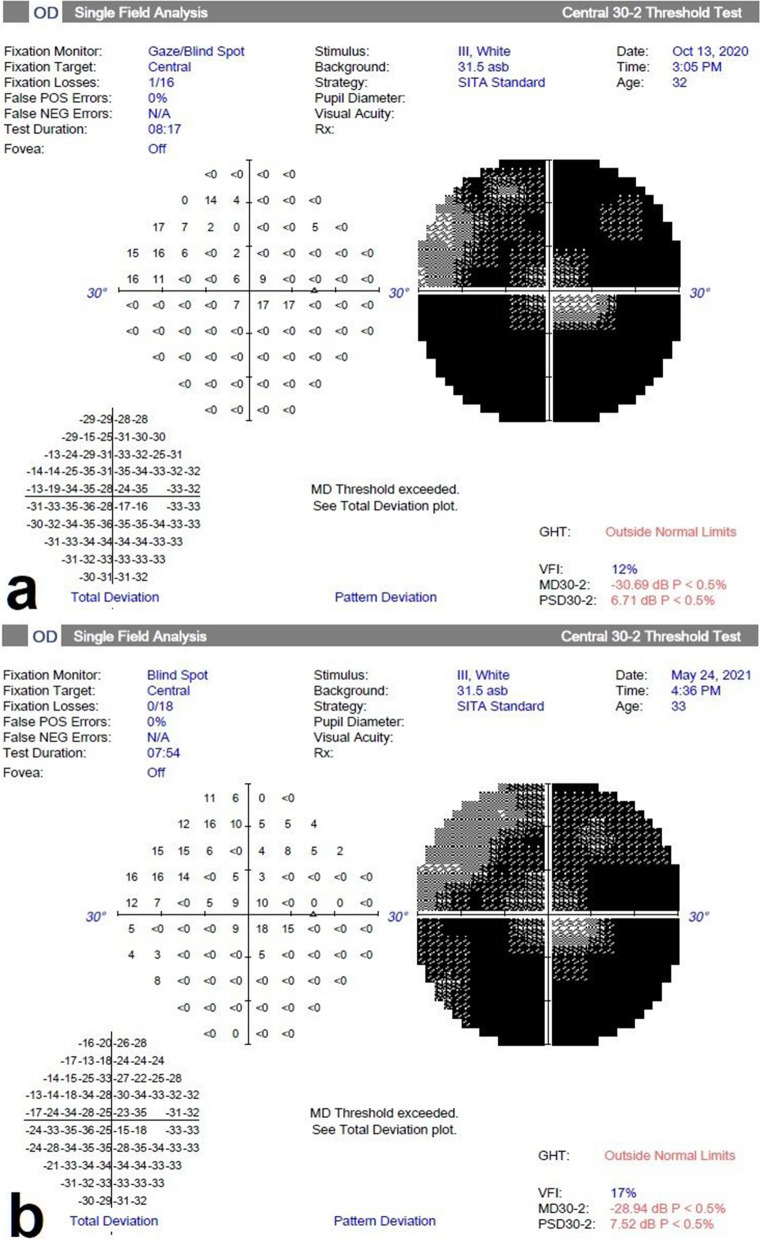
30–2 visual field changes in the spheroidal MSC treatment (Table 1, Patient no. 9). **a** Before the treatment and **b** 6 months later after the treatment. Note the improvement in visual field

**Fig. 5:**
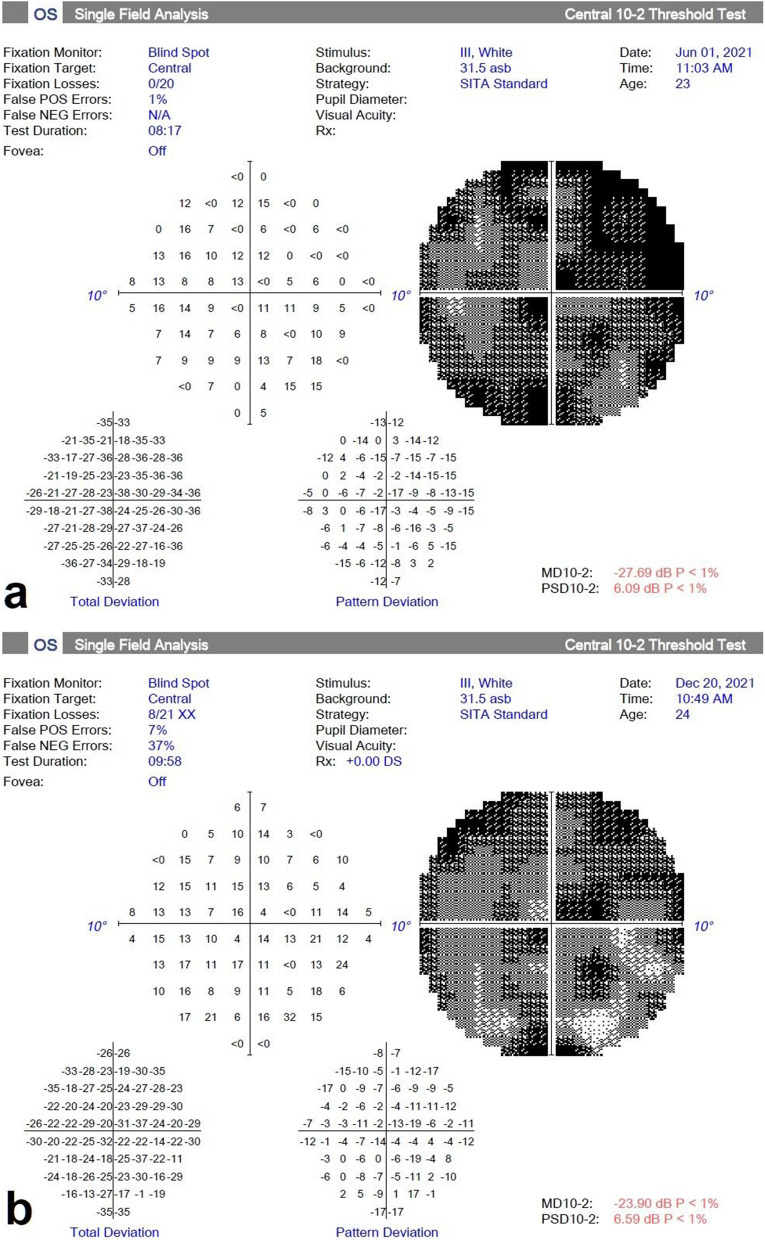
10–2 visual field changes in the spheroidal MSC treatment (Table 1, Patient no. 3). **a** Before the treatment and **b** 6 months later after the treatment. Note the improvement in visual field

The median MD value conducted from treated eyes’ 30–2 VF test was 32.18 (30.37–32.82) at baseline. It was 31.5 (29.11–32.59), 31.63 (19.88–32.42) and 30.58 (27.05–32.46) at the 1st, 3rd and 6th month follow-up examinations. The median MD was significantly improved at the 6th month compared to baseline (*p* = 0.030) (Table 2) (Fig. 4). However, there was no difference in the median PSD values of the 30–2 VF test of the treated eyes (*p* = 0.218). The median MD value conducted from treated eyes’ 10–2 VF test was 27.69 (25.20–34.33) at baseline. It was 26.29 (21.77–34.26), 26.08 (21.02–34.15) and 28.69 (21.59–34.28) at the 1st, 3rd and 6th months, respectively. The median MD value of the 10–2 VF test of the treated eyes was significantly different at the 6th month compared to the baseline (*p* = 0.043). The mean PSD of the 10–2 VF test of the treated eyes was 5.48 (3.01–6.09), 4.71 (2.98–5.96), 4.76 (1.49–5.34) and 4.95 (1.74–6.25) at baseline, 1st, 3rd and 6th month, respectively. PSD value of the 10–2 VF test of the treated eyes was significantly different at the 6th month compared to the 3rd month (Table 2) (Fig. 5). No significant change was observed in MD and PSD of the both 10–2 and 30–2 VF tests of the fellow eyes (*p* > 0.05, for all) (Table 2).

**Fig. 6:**
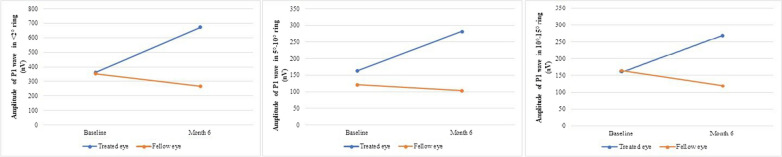
Trends of the amplitudes of P1 waves in < 2°, 5°–10° and 10°–15° rings of treated and fellow eyes during the follow-up

In the mfERG test, the results of 11 patients were analyzed because of poor fixation or artifacts in the test results of the remaining four patients. Table 3 demonstrates the amplitudes of P1 waves of treated and fellow eyes. The amplitudes of P1 waves in < 2°, 5°–10° and 10°–15° rings improved significantly at the postoperative 6th month compared to baseline in treated eyes (*p* = 0.014, *p* = 0.018 and *p* = 0.017, respectively) (Fig. 6). Despite the amplitudes of P1 waves in 2°–5° and > 15° rings being improved, the changes were not statistically significant. No significant changes were observed in the recordings of the fellow eyes at the 6th month compared to baseline (*p* > 0.05, for all). Furthermore, the amplitudes of P1 waves in < 2°, 5°–10° and 10°–15° rings were significantly increased in treated eyes compared to fellow eyes at the postoperative 6th month (*p* = 0.001, *p* = 0.009 and *p* = 0.027, respectively).

**Table 3:** Comparison of mfERG amplitudes of P1 waves at baseline and final examination (6th months) (*n* = 11)

	Ring	Amplitude of P1 wave (nV)		p*
		Preop	Postop. 6th months	
** < 2°**				
Treated eye	Median (IQR)	362.0 (159.0–612.0)	672.0 (564.0–1013.0)	**0.014**
	Mean ± SD	412.7 ± 291.7	830.3 ± 497.9	
Fellow eye	Median (IQR)	352.0 (163.0–600.0)	268.0 (225.0–564.0)	0.509
	Mean ± SD	399.1 ± 254.0	347.7 ± 174.9	
***p***		0.909**	0.001***	
**2°–5°**				
Treated eye	Median (IQR)	227.0 (180.0–297.0)	217.0 (167.0–446.0)	0.101
	Mean ± SD	215.2 ± 86.7	299.0 ± 174.2	
Fellow eye	Median (IQR)	180.0 (100.0–250.0)	200.0 (95.0–386.0)	0.406
	Mean ± SD	219.0 ± 175.0	255.3 ± 195.5	
***p***		0.365***	0.562***	
**5°–10°**				
Treated eye	Median (IQR)	163.0 (111.0–231.0)	282.0 (127.0–313.0)	**0.018**
	Mean ± SD	175.4 ± 86.4	248.3 ± 108.5	
Fellow eye	Median (IQR)	121.0 (75.0–131.0)	103.0 (94.0–198.0)	0.834
	Mean ± SD	129.0 ± 68.5	134.0 ± 75.2	
***p***		0.133***	0.009**	
**10°–15°**				
Treated eye	Median (IQR)	161.0 (92.2–170.0)	269.0 (174.0–295.0)	**0.017**
	Mean ± SD	147.4 ± 65.8	267.4 ± 139.5	
Fellow eye	Median (IQR)	164.0 (99.0–198.0)	120.0 (72.4–240.0)	0.883
	Mean ± SD	154.3 ± 62.0	149.6 ± 85.7	
***p***		0.802**	0.027**	
**> 15°**				
Treated eye	Median (IQR)	83.2 (53.1–183.0)	164.0 (91.0–210.0)	0.152
	Mean ± SD	117.2 ± 78.3	152.3 ± 69.5	
Fellow eye	Median (IQR)	77.4 (57.0–97.2)	100.0 (60.0–112.0)	0.053
	Mean ± SD	73.2 ± 26.1	98.3 ± 43.4	
***p***		0.102**	0.088***	

*mfERG* multifocal electroretinogram, *SD* standard deviation and *IQR* interquartile range

*Paired samples *t*-test

**Independent samples *t*-test

***Mann–Whitney *U*-test

Bold face values represent statistical significance

Implicit times of P1 waves of treated and fellow eyes are depicted in Table 4. There was a statistically significant improvement in implicit times of P1 waves in 10°–15° ring at the postoperative 6th month compared to baseline (*p* = 0.004) in treated eyes. The implicit times of P1 waves in < 2°, 2°–5°, 5°–10° and > 15° rings did not differ after the operation. No significant changes were observed in the recordings of the fellow eyes at the postoperative 6th month compared to baseline (*p* > 0.05, for all).

**Table 4:** Comparison of mfERG implicit times of P1 waves at baseline and final examination (6th month) (*n* = 11)

	Ring	Implicit time of P1 wave (ms)		p*
		Preop	Postop. 6th months	
** < 2°**				
Treated eye	Median (IQR)	51.9 (49.6–55.8)	41.4 (36.1–57.8)	0.163
	Mean ± SD	51.4 ± 9.34	44.8 ± 11.6	
Fellow eye	Median (IQR)	52.9 (38.1–64.3)	43.7 (34.4–65.1)	0.754
	Mean ± SD	51.3 ± 14.4	49.5 ± 16.6	
***p***		0.989**	0.0453**	
**2°–5°**				
Treated eye	Median (IQR)	53.6 (43.6–58.2)	43.1 (34.7–46.6)	0.073
	Mean ± SD	51.1 ± 10.3	43.9 ± 9.5	
Fellow eye	Median (IQR)	42.2 (40.6–44.6)	44.3 (32.4–65.5)	0.645
	Mean ± SD	44.1 ± 8.9	46.1 ± 15.9	
***p***		0.065***	0.700**	
**5°–10°**				
Treated eye	Median (IQR)	43.3 (37.9–61.3)	42.2 (32.3–54.6)	0.572
	Mean ± SD	49.9 ± 14.3	46.6 ± 14.8^a^	
Fellow eye	Median (IQR)	53.9 (42.2–69.0)	70.0 (47.7–72.3)	0.281
	Mean ± SD	53.8 ± 14.4	60.6 ± 17.1	
***p***		0.531**	0.088***	
**10°–15°**				
Treated eye	Median (IQR)	65.2 (47.2–70.0)	49.6 (35.6–59.5)	**0.004**
	Mean ± SD	59.2 ± 13.6	47.2 ± 12.2	
Fellow eye	Median (IQR)	47.2 (64.3–67.7)	60.0 (40.4–67.9)	0.120
	Mean ± SD	50.5 ± 16.4	57.7 ± 14.9	
***p***		0.192**	0.087**	
**> 15°**				
Treated eye	Median (IQR)	52.3 (53.1–183.0)	57.3 (43.2–67.5)	0.515
	Mean ± SD	51.0 ± 10.3	54.6 ± 13.2	
Fellow eye	Median (IQR)	52.3 (40.8–63.2)	53.6 (40.0–65.0)	0.952
	Mean ± SD	53.4 ± 11.3	53.1 ± 16.6	
***p***		0.605**	0.819**	

*mfERG* multifocal electroretinogram, *SD* standard deviation and *IQR* interquartile range

*Paired samples *t*-test

**Independent samples *t*-test

***Mann–Whitney *U*-test

Bold face value represents statistically significant improvement in implicit time of P1 waves in 10°–15° rings postoperatively

We did not observe any postoperative complications, side effects or adverse effects during the follow-up period.

## Discussion

RP is an inherited retinal degeneration that causes progressive visual loss. More than 150 genes were found to be related to the disease, and genetic tests of the patients with similar clinical appearance may still show new unidentified genes [1]. Most of these genes are involved in phototransduction, cell trafficking, outer segment membrane structure, neuronal or immune response, rhodopsin recycling pathways or glucose metabolism [2, 28, 29]. These metabolic pathways play fundamental roles in the function and maintenance of the photoreceptor cells. Change in the photoreceptor metabolism and structure triggers cell death [30]. Additionally, mitochondria of the photoreceptor inner segments are also affected, and they lack antioxidant defense maintained by superoxide dismutase, glutathione dismutase and catalase in patients with RP [31, 32]. The combination of these processes leads to apoptosis, regulated necrosis and autophagy, which result in progressive loss of the photoreceptors and progressive loss of vision.

Mesenchymal stem cells (MSCs) can secrete paracrine factors that affect the neuronal microenvironment. These factors induce proliferation and differentiation in the surrounding cells of the tissue in which they were implanted. They also produce chemo-attractants that stimulate the migration of the cells necessary for tissue healing. The secreted therapeutic factors are composed of growth factors and cytokines, vesicular portion of extracellular micro-vesicles and mitochondria [33]. Growth factors and cytokines cause immune modulation, angiogenesis, anti-apoptosis, anti-oxidation, cell migration and stimulation. The main growth factors and cytokines that mesenchymal stem cells produce are basic fibroblast growth factor, vascular endothelial growth factor, macrophage colony-stimulating factor, placental growth factor, transforming growth factor-beta, insulin-like growth factor-1, interleukin, angiogenin, ciliary neurotrophic factor, brain-derived growth factor and glial cell-derived growth factor [34]. Micro-vesicles contain bioactive molecules, RNA, microRNA, lipids and proteins for intercellular communication, and they regulate the metabolism of the retinal cells [35]. Finally, mitochondria affect the healing process by increasing the activity of the respiratory chain complex and ATP levels [36].

The previous studies showed the regenerative effect of mesenchymal stem cells in retinal diseases. Since studies using intravitreal injection or subretinal injection method for MSCs implantation resulted in side effects and complications [37, 38], sub-tenon or suprachoroidal route was recommended [3–6, 39, 40]. Özmert and Aslan have chosen the sub-tenon administration method for patients with RP. The eyes completing 1-year follow-up after the treatment showed significant improvement in visual acuity, visual field and ERG [6]. Limoli et al. [39] created a new technique for suprachoroidal implantation of stem cells. They created a scleral flap that they could see the reflex of the choroid in the flap’s bed. Then, they mobilized the fat pad near the inferior oblique muscle and placed it into this bed. After suturing the flap back, they injected the MSCs inside the fat pad. By this way, the investigators created a passage for the regenerative factors of the MSCs without placing them into the eye. First, they used this technique in patients with dry age-related macular degeneration. They followed the patients for 6 months, and they found that BCVA was improved, and microperimetry responses were increased in the treatment group. They also performed this technique in patients with RP [40]. The same technique was used in patients with RP and age-related macular degeneration by Kahraman et al. [3]. They evaluated BCVA, visual field and mfERG recordings of the patients with age-related macular degeneration, and they found improvements in these parameters at the 1st year of the follow-up. In patients with RP, they also found significant improvement in BCVA, visual field and mfERG recordings [4]. None of these studies report any complications or side effects with this treatment [3–6, 39, 40].

Limoli retinal restoration technique is a well-designed method for delivering mesenchymal stem cells. The adipose pedicle placed in the scleral bed maintains a safe and nutrient environment for the mesenchymal stem cells, additionally, being able to reintroduce PRP in the following period improve the efficacy of the method, and increases the duration of the mesenchymal secretome [39]. In our study, we used a matrix instead of a fat pedicle for two reasons. First, mesenchymal stem cells spheroids are too large to inject, they have to be placed. It is easier to place them in a solid bed. The second and more important reason is the characteristics of the extracellular matrix (ECM). It has been shown that when the stem cells are placed in phosphate-buffered saline (PBS), they lose their correct cell/ECM connections. This results in a form of apoptosis, termed anoikis [41]. ECM provides a scaffold for the stem cells increasing their viability. It has been reported that encapsulating stem cells in an ECM creates a pro-regenerative environment, and paracrine effects of the stem cells could be optimized [42]. Additionally, ECM may deliver numerous soluble and immobilized factors that could elevate the therapeutic effects of the stem cells [43].

MSC preparation as spheroids has been shown to have improved anti-inflammatory, regenerative and reparative effects [41–45]. When MSCs form spheroids, they create a microenvironment that improves their survival. In an animal study, it has been demonstrated that spheroidal stem cells remained for 14 days in the tissues. On the other hand, the persistence of suspension MSC injections is limited to 7 days in same study [46]. The microenvironment upregulates the potential of each MSC and strengthens its secretion. As a result, paracrine secretion of angiogenic, antitumorigenic, anti-inflammatory and immunomodulatory is enhanced, differentiation potentials are increased and replicative senescence is delayed in this microenvironment [47]. The previous studies demonstrated the regenerative effect of spheroidal MSCs in cardiomyocytes, neural cells, cartilage and wound healing [4, 48–50]. In the light of these findings, it could be possible to propose that the improved spheroidal MSCs may also be more effective in regenerating and protecting the retinal cells. In line with that idea, our study demonstrated a significant improvement in visual acuity and visual fields of the patients with RP with spheroidal MSCs. To the best of our knowledge, the present study is the first to use spheroidal MSCs in the treatment of RP. Further studies that would show the duration of these effects should be conducted. Methods for re-implantation or PRP re-injection (like in LRRT) should also be investigated.

In phase III clinical trial of suprachoroidal implantation of umbilical cord-derived MSC in RP, suprachoroidal implantation, which we modified using spheroids as described above, Kahraman and Öner [4] reported visual acuity improvement in 46% of the patients (*p* < 0.05), stabilization in 42% and worsening in 12% in 6 months follow-up period. However, our results demonstrated visual acuity improvement in 80% of the patients (*p* < 0.001) and stabilization in 20% of the patients. None of our patients had worsening in visual acuity during the follow-up period. Similar to our study, they also showed significant improvement in 30/2 visual field testing of the patients. Moreover, 10/2 visual field testing showed significant improvement in the current study. mfERG tests in Kahraman and Öner study [4] revealed statistically significant improvement in P1 amplitudes in the central rings (< 2° and 2°–5°) and a slight decrease in the peripheral rings. The current study conducted with patients with relatively good visual acuity revealed improvement in P1 amplitudes in all mfERG rings, but the improvements in < 2°, 5°–10° and 10°–15° rings were statistically significant. These findings are in line with Limoli et al. study conducted on RP patients which found that patients with foveal thickness > 190 microns had markedly good BCVA [40]. Additionally, further studies that compare the effects of spheroidal MCSs with sub-tenon or suprachoroidal injection of non-spheroidal MSCs should be carried out.

The small sample size and short duration of follow-up can be considered limitations of our study. The second limitation was that there was not any study in the literature using spheroidal MSCs in the treatment of RP, and thus, the results of our study could not be supported and compared. Additionally, the genetic mutation analyses of the patients were variable, and we could not make a comment related with genetic factor and treatment response. Further studies with a larger sample size and longer follow-up can be performed to support our findings.

## Conclusion

RP is characterized by progressive loss of photoreceptors and consequently loss of visual acuity. Current treatment modalities are not able to treat the disease. Our results provide clear evidence that MSCs are shown to have regenerative effects in patients with RP. Moreover, this is the first study to show visual improvement with mesenchymal stem cell application in RP patients with relatively good visual acuity. Spheroidal MSCs with enhanced effects may be more successful in preventing apoptosis and improving cellular restoration.

## Data Availability

Data available on request.

## Abbreviations

RP: Retinitis pigmentosa
RPE: Retinal pigment epithelium
MSC: Mesenchymal stem cell
BCVA: Best-corrected visual acuity
MD: Mean deviation
PSD: Pattern standard deviation
VF: Visual field
ISCEV: International Society for Clinical Electrophysiology of Vision
logMAR: Logarithm of the minimal angle of resolution
mfERG: Multifocal electroretinography
PRP: Platelet-rich plasma
MSAs: Mean signal amplitudes
ECM: Extracellular matrix
PBS: Phosphate-buffered saline
